# Curative or Conservative Approaches: A Systematic Review of Surgical and Nonsurgical Treatments for Basal Cell Carcinoma

**DOI:** 10.7759/cureus.95556

**Published:** 2025-10-28

**Authors:** Christine Suryani Novelita Sutrisno, Desy Hinda Pramita, Ita Puspita Dewi

**Affiliations:** 1 Department of Dermatology Venereology, Dr. Mohamad Soewandhie Regional General Hospital, Surabaya, IDN

**Keywords:** basal cell carcinoma, conservative management, nonsurgical treatment, surgical excision, systematic review

## Abstract

Basal cell carcinoma (BCC) is the predominant kind of skin cancer globally, with a consistently increasing prevalence attributed to heightened ultraviolet (UV) exposure, aging demographics, and enhanced diagnostic techniques. Surgical excision is considered the gold standard for BCC treatment; however, various nonsurgical alternatives, such as topical therapies, photodynamic therapy (PDT), cryotherapy, and ablative techniques, are increasingly utilized, especially in patients with low-risk lesions or contraindications to surgery. Comparative assessments of various modalities are crucial for informing evidence-based, patient-centered treatment choices. To comprehensively evaluate the effectiveness, recurrence rates, cosmetic outcomes, safety profiles, and clinical applicability of surgical and nonsurgical treatment options for basal cell carcinoma, this systematic review was conducted in accordance with Preferred Reporting Items for Systematic Reviews and Meta-Analyses (PRISMA) guidelines. Extensive literature searches were performed in PubMed, ScienceDirect, and the Cochrane Library up to May 29, 2025, using keywords related to "surgical excision," "basal cell carcinoma," and associated comparative terms. Studies were eligible if they involved human participants and directly compared surgical and nonsurgical interventions. The risk of bias was evaluated with the Risk of Bias In Non-randomized Studies of Interventions (ROBINS-I) and Risk of Bias 2 (RoB-2) instruments. Eleven papers fulfilled the inclusion criteria for qualitative synthesis. Surgical excision consistently exhibited enhanced long-term effectiveness and reduced recurrence rates in comparison to nonsurgical interventions. Nonetheless, nonsurgical approaches, especially topical imiquimod and PDT, demonstrated similar efficacy in addressing superficial or low-risk lesions while providing markedly superior esthetic results. In older or medically challenged individuals, conservative therapies were well-tolerated and clinically efficacious. Nonetheless, recurrence risks were often elevated with nonsurgical methods, especially in nodular or deeper tumors. The findings confirm that surgical excision is the ultimate therapy for basal cell carcinoma, particularly in high-risk or aggressive cases. Nevertheless, nonsurgical treatments provide significant advantages regarding esthetics, tolerability, and patient comfort, rendering them appropriate options in some instances. The variability of studies and the scarcity of long-term data highlight the necessity for more research utilizing standardized metrics and prolonged follow-up durations. In conclusion, surgical excision is the most successful and dependable treatment for basal cell carcinoma; nonetheless, nonsurgical alternatives are significant for specific patient populations. Customized treatment planning, guided by lesion attributes, patient comorbidities, and esthetic factors, is crucial for enhancing results and facilitating collaborative decision-making in clinical practice.

## Introduction and background

Basal cell carcinoma (BCC) is the most prevalent kind of skin cancer [1]. Owing to its low death rate, cancer registries in most nations exclude data on BCC; yet, data from insurance registries and government statistics estimate the yearly incidence of BCC in the United States to be 4.3 million [1]. The Caucasian demographic exhibits a significantly elevated incidence of BCC. The prevalence of BCC is inversely related to a nation's geographic latitude and the pigmentation of its population [2]. Comparable incidence rates have been identified in Canada, Europe, and Asia, but Australia exhibits the highest prevalence worldwide. While the incidence trend in Australia seems to have stabilized, the rate continues to rise throughout all other continents, including South America and Asia. A comprehensive review by Perera et al. indicated that Australia had the highest prevalence of nonmelanoma skin cancer globally [3]. The prevalence was greater in males compared to females and higher for basal cell carcinoma than for squamous cell carcinoma. The incidence varied between Australian states, with Queensland exhibiting the greatest rate. The assertive Slip! Slop! Slap! campaign has markedly impacted skin cancer prevention efforts, demonstrating considerable advantages [4]. In Europe, the incidence has increased by 5% annually over the past decade, in contrast to around 2% in the United States. This epidemiological trend is anticipated to persist in the near future due to enhanced diagnostic capabilities and an aging population with a history of ultraviolet (UV) exposure [1-3]. The prevalence of BCC significantly escalates beyond the age of 40 years; nevertheless, there has recently been a rise in cases among younger individuals, especially women, due to heightened UV exposure from both natural and artificial sources [5].

The patched/hedgehog intracellular signaling system regulates cell proliferation, and its persistent activation facilitates the development of BCC [6]. Inactivating mutations in patched homolog 1 (PTCH1) and activating mutations in smoothened (SMO) are the most common mutations, leading to aberrant activation of the hedgehog pathway and tumorigenesis. A tiny number of BCCs have a loss-of-function mutation in the SUFU gene, which serves as a negative regulator of the hedgehog signaling pathway [7]. Ultraviolet-specific mutations in the p53 tumor suppressor gene, seen in 50% of basal cell carcinomas, represent another common alteration [7].

Fitzpatrick skin types I and II exhibit a heightened risk of developing BCCs, with a lifetime risk of 30%. Individuals with light eye color, freckles, and red hair exhibit an increased susceptibility to BCC [7]. The primary environmental risk factor is exposure to ultraviolet radiation. Childhood sunburns, familial predisposition, photosensitizing medications, ionizing radiation, tanning bed usage, chronic immunosuppression, and exposure to carcinogenic agents, especially arsenic, constitute significant risk factors [8,9]. The onset of BCC is closely associated with infancy and significant, sporadic sun exposure [10,11].

The initial step in diagnosing BCC is inspection, succeeded by dermoscopy, with confirmation obtained by biopsy and histopathologic analysis. Capturing images of the lesion is crucial for enabling the surgeon to accurately identify the location during the final operation. Incorrect site-definitive techniques are the most significant problem in this approach [12]. A skin biopsy remains essential to confirm the clinical assessment. BCC is histologically characterized by the proliferation of uniform basaloid cells featuring hyperchromatic nuclei and scant poorly defined cytoplasm, along with peripheral palisading and retraction artifacts [13]. Although basaloid cells have physical similarities to epidermal basal cells, their behavior resembles that of follicular germinative cells [14,15]. BCC presents in several clinicopathologic forms, including nodular, infiltrative, fibroepithelial, morpheaform, and superficial, each exhibiting distinct clinicopathologic features. Micronodular and basosquamous basal cell carcinomas are the two most significant histological categories, and treatment approaches may differ based on the BCC types.

Although BCC is the most common kind of skin cancer worldwide, it remains challenging to identify the best suitable treatment option for each patient. Surgical excision, encompassing ordinary excision and Mohs micrographic surgery, is esteemed as the gold standard owing to its elevated cure rates and little recurrence [16]. Nonetheless, it may not be appropriate for all patients, especially those with comorbidities, elderly individuals, or those with tumors situated in cosmetically or functionally sensitive regions, such as the face. In certain instances, nonsurgical alternatives, such as topical treatments (e.g., imiquimod, 5-fluorouracil), photodynamic therapy, cryotherapy, and radiotherapy, are being increasingly employed. These therapies are frequently less intrusive, more tolerable, and favored by patients who prioritize esthetic results. Nonetheless, the trade-offs among convenience, cost, long-term effectiveness, and recurrence risk remain ambiguous and are reported inconsistently in the literature. As clinical practice evolves to prioritize patient-centered care and collaborative decision-making, comprehending the comparative efficacy of treatment modalities is essential.

The rising prevalence of BCC, aggravated by aging demographics, heightened sun exposure, and enhanced early detection, imposes a considerable strain on global healthcare systems. As a result, physicians encounter increasing pressure to provide treatment that is both effective and resource-efficient, while also aligning with the specific needs and values of individual patients. Although current guidelines include broad recommendations, they frequently lack comprehensive, evidence-based comparisons between surgical and nonsurgical interventions across diverse patient and tumor attributes. Moreover, the existing corpus of research is disjointed, with studies differing significantly in design, assessed outcomes, and period of follow-up. This highlights the pressing necessity for a thorough synthesis of existing research to guide clinical guidelines, enhance patient outcomes, and facilitate cost-effective healthcare delivery. This systematic review seeks to address the existing knowledge gap by assessing and contrasting the efficacy, safety, cosmetic results, recurrence rates, and patient satisfaction linked to surgical and nonsurgical management of BCC, thereby facilitating more informed and nuanced treatment decisions.

## Review

Methods

Search Strategy

This systematic review was conducted and documented in accordance with Preferred Reporting Items for Systematic Reviews and Meta-Analyses (PRISMA) guidelines. A comprehensive literature review was conducted using PubMed, ScienceDirect, and the Cochrane Library, completed on May 29, 2025. For this literature study, we utilized the following keywords: "Surgical Excision" AND Basal Cell Carcinoma AND ("Comparative" OR "versus"). This technique will encompass publications, including pertinent titles and abstracts, to enable comprehensive examination and subsequent quantitative and qualitative analysis.

Inclusion and Exclusion Criteria

**Figure 1:**
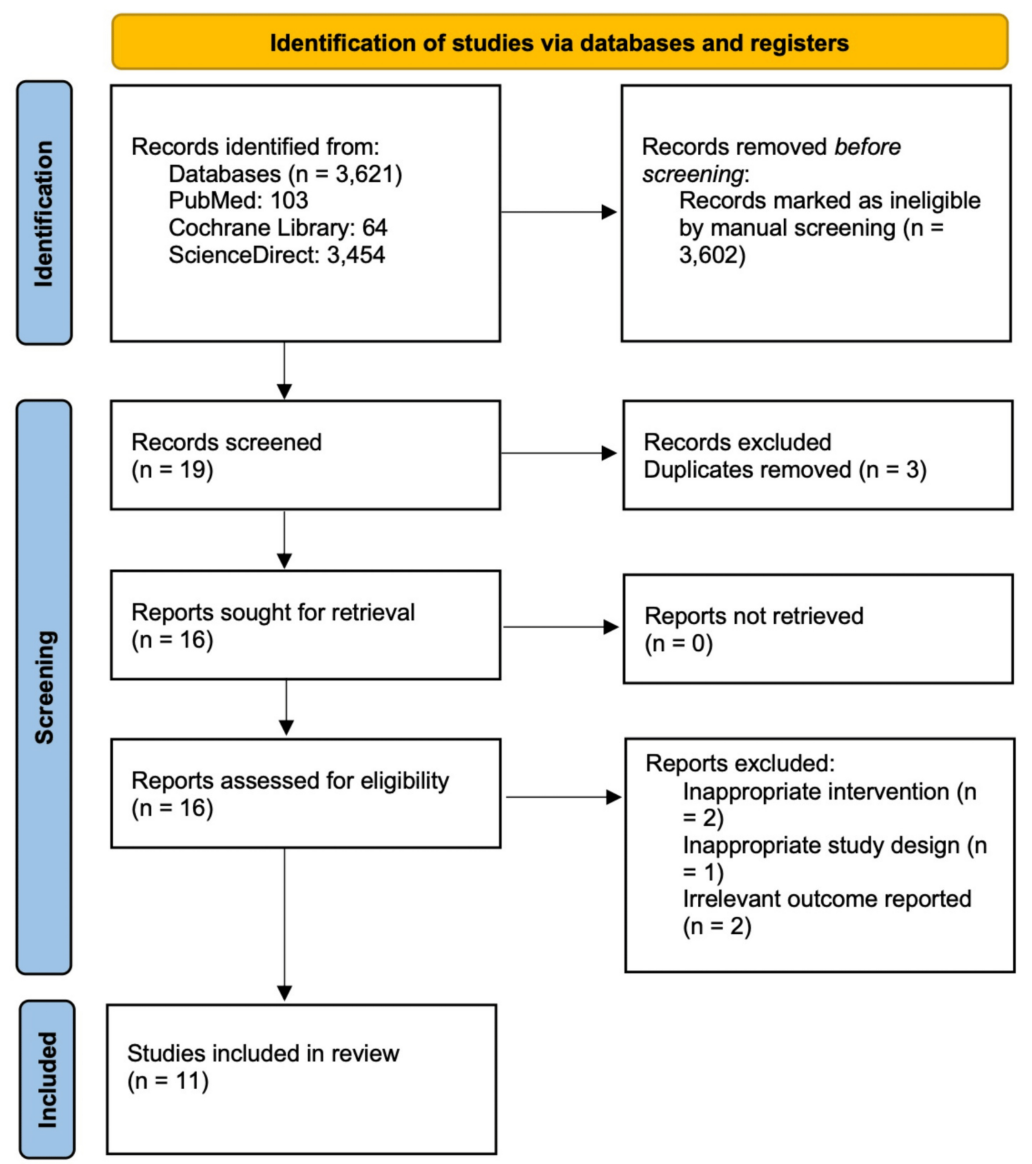
PRISMA 2020 flow diagram showing the number of records identified, screened, excluded, and included in the systematic review. PRISMA: Preferred Reporting Items for Systematic Reviews and Meta-Analyses

We utilized the following criteria to determine the eligibility of studies for inclusion: (1) comparative research on outcomes of surgical versus nonsurgical therapy in basal cell carcinoma patients; and (2) studies using human subjects. The following exclusion criteria included publications having inaccessible complete texts, along with studies demonstrating design, intervention, or outcome deficiencies. Figure 1 illustrates the number of records identified, screened, excluded, and included at each stage of the systematic review process, following the PRISMA 2020 guidelines.

Data Extraction and Risk of Bias Assessment

**Figure 2:**
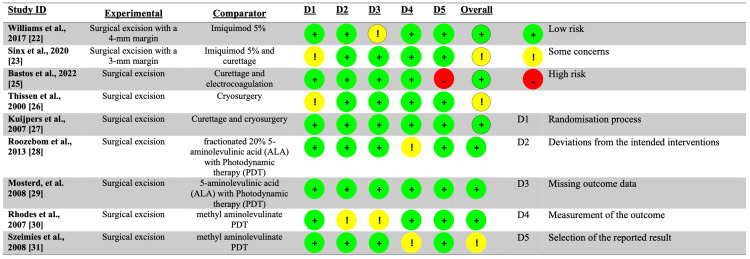
Risk of bias assessment using the RoB-2 tool. Risk of bias assessment using the RoB-2 tool, which evaluates five domains (D1-D5) as follows: D1 represents bias arising from the randomization process, D2 reflects bias due to deviations from the intended interventions, D3 concerns bias due to missing outcome data, D4 indicates bias in the measurement of the outcome, and D5 refers to bias in the selection of the reported result. The overall risk of bias was judged as low, some concerns, or high according to the cumulative assessment across all domains. RoB-2: Risk of Bias 2

**Figure 3:**
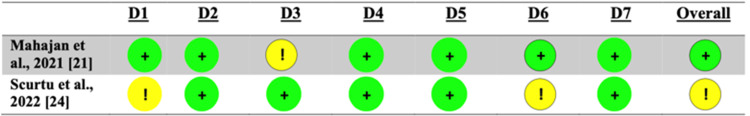
Risk of bias assessment using the ROBINS-I tool. Risk of bias assessment using the ROBINS-I tool, which evaluates seven domains (D1-D7) as follows: D1 represents bias due to confounding, D2 reflects bias in the selection of participants into the study, D3 concerns bias in the classification of interventions, D4 addresses bias due to deviations from intended interventions, D5 relates to bias due to missing data, D6 indicates bias in the measurement of outcomes, and D7 refers to bias in the selection of the reported result. The overall risk of bias was categorized as low, some concerns, or high based on the combined domain assessments. ROBINS-I: Risk of Bias In Non-randomized Studies of Interventions

Following that, we extracted data from the selected publications. Furthermore, the Consolidated Standards of Reporting Trials (CONSORT) or Strengthening the Reporting of Observational Studies in Epidemiology (STROBE) criteria were used to assess the quality of the papers [17,18]. All of the reviewers collaborated to reach a consensus on the work's quality. To assess the possibility of bias, the Risk of Bias in Non-randomized Studies of Interventions (ROBINS-I) or Risk of Bias 2 (RoB-2) instruments created by Cochrane were utilized (Figures 2, 3) [19,20].

Results

Eleven papers fulfilled the inclusion criteria and were incorporated into the qualitative synthesis, consisting of both randomized controlled trials (RCTs) and observational studies (Table 1). The studies were performed in several geographic regions, including India, the United Kingdom, the Netherlands, Romania, Brazil, and Germany. The sample sizes exhibited significant variation, spanning from a minimum of 27 individuals to over 380 in bigger studies. All included studies concentrated on histopathologically or clinically verified cases of BCC and compared surgical excision, typically conducted with a 3 or 4 mm margin, with various nonsurgical interventions, including topical agents, cryotherapy, photodynamic therapy (PDT), or curettage.

**Table 1:** Characteristics and results of the included studies. BCC: basal cell carcinoma; SE: surgical excision; PDT: photodynamic therapy; RCT: randomized controlled trial; ALA: aminolevulinic acid; MAL: methyl aminolevulinate

Studies	Study design	Population	Study location	Sample size	Surgical management	Nonsurgical management	Results
Mahajan et al. (2021) [21]	Retrospective cohort	Histopathologically confirmed BCC	India	51 surgical, 17 nonsurgical	Surgical excision with a 4 mm margin	Imiquimod 5%, cryotherapy, radiofrequency, or radiotherapy	Surgical excision and repair yielded superior results compared to nonsurgical and ablative interventions. Treatment failures and adverse effects were prevalent with nonsurgical and ablative interventions. The recurrence rate was minimal.
Williams et al. (2017) [22]	RCT	Histopathologically confirmed BCC	UK	177 surgical, 206 nonsurgical	Surgical excision with a 4 mm margin	Imiquimod 5%	The study found that imiquimod has higher success rates than surgery, with a relative risk of 0.84, indicating that surgery is more effective for early-responsive lesions, despite the majority of imiquimod therapy failures occurring within the first year.
Sinx et al. (2020) [23]	RCT	Histopathologically confirmed BCC	Netherlands	72 surgical, 73 nonsurgical	Surgical excision with a 3 mm margin	Imiquimod 5% and curettage	Considering the sustained success of curettage and imiquimod cream, together with the sluggish development pattern of nodular basal cell carcinoma (nBCC), these treatments may remain a viable choice to mitigate the excessive use of excisional procedures. Nonetheless, it cannot supplant surgical excision.
Scurtu et al. (2022) [24]	Quasi-experimental study	Histopathologically confirmed BCC	Romania	46 surgical, 103 nonsurgical	Surgical excision	Combination of ablative CO_2_ laser, cryosurgery, and topical 5-fluorouracil and imiquimod	This conservative approach is appropriate for elderly individuals and people with comorbidities, demonstrating comparable efficacy to surgery for clearance, recurrence rates, and local adverse events, while exhibiting superior esthetic results.
Bastos (2022) [25]	RCT	Clinically evident BCC	Brazil	61 surgical, 55 nonsurgical	Surgical excision	Curettage and electrocoagulation	The evaluation conducted three months post-procedure revealed no significant differences in treatment satisfaction, local care difficulties, symptoms, or surgical complications, although there were slight yet significant differences concerning scar appearance.
Thissen et al. (2000) [26]	RCT	Histopathologically confirmed BCC	Netherlands	48 surgical, 48 nonsurgical	Surgical excision	Cryosurgery	In general, cosmetic outcomes following excision are superior to those resulting from cryosurgery.
Kuijpers et al. (2007) [27]	RCT	Histopathologically confirmed BCC	Netherlands	49 surgical, 51 nonsurgical	Surgical excision	Curettage and cryosurgery	A clinically significant difference appears to exist between the recurrence rates of surgical excision and curettage and cryosurgery; however, this difference did not achieve statistical significance.
Roozeboom et al. (2013) [28]	RCT	Histopathologically confirmed BCC	Netherlands	88 surgical, 83 nonsurgical	Surgical excision	Fractionated 20% 5-aminolevulinic acid (ALA) with photodynamic therapy (PDT)	The five-year cumulative likelihood of recurrence following surgical excision is inferior to that after fractionated ALA-PDT with preceding debulking. While surgical removal is the definitive treatment, photodynamic therapy may serve as an option for inoperable individuals with thin (≤0.7 mm) nodular basal cell carcinoma.
Mosterd et al. (2008) [29]	RCT	Histopathologically confirmed BCC	Netherlands	16 surgical, 11 nonsurgical	Surgical excision	5-aminolevulinic acid (ALA) with photodynamic therapy (PDT)	SE had much greater efficacy compared to treatment with fractionated illumination ALA-PDT.
Rhodes et al. (2007) [30]	RCT	Histopathologically confirmed BCC	UK	52 surgical, 53 nonsurgical	Surgical excision	Methyl aminolevulinate PDT	Long-term follow-up demonstrates the greater effectiveness of surgery compared to methyl aminolevulinate photodynamic therapy in nodular basal cell carcinoma. Methyl aminolevulinate PDT is an effective therapy for this condition and demonstrates a more advantageous esthetic result.
Szeimies et al. (2008) [31]	RCT	Histopathologically confirmed BCC	Germany	96 surgical, 100 nonsurgical	Surgical excision	Methyl aminolevulinate PDT	MAL-PDT provides comparable effectiveness and superior cosmetic results compared to basic excision surgery in the treatment of BCC.

Mahajan et al. conducted research in India that demonstrated the superiority of surgical excision compared to a mix of nonsurgical techniques, including imiquimod, cryotherapy, and radiation, noting reduced recurrence rates and fewer adverse events in the surgical cohort [21]. In a substantial RCT conducted in the United Kingdom, Williams et al. indicated that although imiquimod exhibited comparatively high success rates, surgical excision proved to be more efficacious, especially for lesions that responded early [22]. This discovery highlights the significance of lesion attributes and reactivity in establishing the most effective therapy.

Sinx et al. and Scurtu et al. assessed combinations of topical and ablative treatments [23,24]. Sinx et al. noted that the combination of imiquimod and curettage offered a feasible option for nodular BCC due to its gradual development; however, surgery continued to be the ultimate therapy [23]. In a quasi-experimental study conducted in Romania, Scurtu et al. underscored the importance of conservative methods, such as CO₂ laser, cryosurgery, and topical agents, for older patients and individuals with comorbidities, noting comparable efficacy and enhanced esthetic results relative to surgical interventions [24].

Bastos et al. and Kuijpers et al. evaluated curettage-based methodologies [25,27]. Bastos et al. observed no substantial disparities in treatment satisfaction or problems between the surgical and nonsurgical cohorts, but minor variations in scar outcomes were noted. Kuijpers et al. observed a clinically significant albeit statistically insignificant disparity in recurrence rates between surgical intervention and the combination of curettage and cryotherapy, indicating the possible efficacy of less invasive techniques in specific clinical scenarios [25,27].

Numerous studies have contrasted surgical excision with photodynamic treatment (PDT). Roozeboom et al. demonstrated that surgical excision exhibited a reduced five-year recurrence rate relative to fractionated 5-aminolevulinic acid (ALA)-PDT, but PDT continued to be a feasible alternative for thin, inoperable tumors [28]. Mosterd et al. validated the greater effectiveness of surgery compared to ALA-PDT, whereas Rhodes et al. and Szeimies et al. contrasted surgery with methyl aminolevulinate (MAL)-PDT [29-31]. While surgery has shown superior long-term effectiveness, MAL-PDT provided markedly improved esthetic results, rendering it an appropriate option for some situations.

The results consistently supported surgical excision as the definitive standard for BCC therapy, especially for long-term effectiveness and recurrence prevention. Nonsurgical therapies, including imiquimod, cryotherapy, and photodynamic therapy, have shown efficacy, particularly in individuals for whom surgery was contraindicated or undesired owing to esthetic or health considerations. Numerous studies highlighted patient-centered criteria, including tolerability, esthetics, and treatment convenience, in informing personalized therapy selections.

Despite their diversity, the included studies collectively highlight the intricate trade-offs among effectiveness, safety, and esthetic outcomes in the management of BCC. Although surgery is the most efficacious method, the advancing role of nonsurgical treatments, especially in low-risk or cosmetically sensitive situations, advocates for a more individualized strategy in managing BCC, harmonizing clinical results with patient preferences and comorbidities.

Discussion

This systematic review analyzed the comparative results of surgical versus nonsurgical interventions for BCC across 11 studies employing diverse methodologies and patient demographics. The research encompassed randomized controlled trials (RCTs), retrospective cohort studies, and quasi-experimental designs carried out in several geographical locations, including India, the United Kingdom, the Netherlands, Germany, Romania, and Brazil. The procedures contrasted surgical excision, mainly with clinical margins of 3-4 mm, against nonsurgical methods, such as topical medications (e.g., imiquimod, 5-fluorouracil), photodynamic therapy (PDT), cryotherapy, curettage, electrocoagulation, and laser ablation. The data collectively confirm that surgical excision is the gold standard for final therapy of BCC, especially for long-term tumor elimination and recurrence prevention [16,32]. Nonsurgical therapies have shown significant success, particularly in certain groups, such as the elderly or patients with comorbidities, and frequently resulted in enhanced esthetic outcomes.

Surgical excision has shown consistently low recurrence rates throughout the investigations, attributable to its thorough tumor removal and histological verification of margin clearing [16,32]. Research conducted by Mahajan et al., Williams et al., and Roozeboom et al. indicates that excision surpassed nonsurgical methods for long-term tumor management [21,22,28]. This discovery corroborates clinical guidelines advocating for surgical excision, especially in cases of nodular, infiltrative, or recurring BCCs, when full tumor elimination is essential. Histological study of removed tissue facilitates precise staging and risk assessment, which nonsurgical approaches cannot do. Standard surgical margins of 3-4 mm are adequate for low-risk lesions, minimizing tissue excision while maintaining therapy efficacy.

Conversely, topical therapies like imiquimod and 5-fluorouracil function via processes dependent on the host's immune response and localized cytotoxic effects, respectively. Imiquimod activates toll-like receptor 7, inducing the synthesis of pro-inflammatory cytokines, including interferon-alpha and tumor necrosis factor-alpha, resulting in localized immune-mediated eradication of cancer cells. This method is minimally invasive and maintains tissue structure, although its effectiveness varies depending on the host's immunological competency and tumor depth. Research by Williams et al. and Sinx et al. indicated modest efficacy of imiquimod, especially in superficial BCC, but elevated treatment failure rates were observed in nodular or more substantial lesions [23,33,34]. The requirement for extended use and localized skin responses, including erythema, erosion, and discomfort, may diminish patient adherence.

Photodynamic treatment (PDT) was a significant nonsurgical approach assessed in several studies, including those conducted by Roozeboom et al., Rhodes et al., and Szeimies et al. Photodynamic therapy (PDT) is the topical administration of a photosensitizer, such as 5-aminolevulinic acid (ALA) or methyl aminolevulinate (MAL), which selectively accumulates in neoplastic cells [28,30,31]. When activated by specified light wavelengths, the photosensitizer produces reactive oxygen species that selectively promote tumor cell death. This precise process minimizes harm to adjacent healthy tissue, yielding advantageous esthetic effects. PDT had significant effectiveness for superficial and thin nodular BCC; however, its limits were apparent in deeper lesions due to inadequate light penetration and inefficient photosensitizer absorption [34]. Surgical excision demonstrated superior efficacy for long-term clearance, particularly in lesions above 0.7 mm in depth.

Nevertheless, cosmetic results preferred nonsurgical interventions. PDT, imiquimod, and cryotherapy produced little scarring, superior preservation of skin texture, and reduced pigmentary changes in comparison to surgical excision. This was particularly evident in studies such as those by Scurtu et al. and Szeimies et al., in which patient satisfaction with aesthetic outcomes was considerably higher in the nonsurgical cohorts [24,31]. Conversely, despite meticulous execution, surgical excision inherently poses risks of scarring and contour abnormalities, especially in cosmetically sensitive regions, such as the face or neck. This is a crucial factor for people who value cosmetic outcomes or are apprehensive about post-operative scars.

Patient-centered factors significantly influenced the decision to undergo therapy. Nonsurgical techniques provided excellent and safer options for elderly patients, persons with multiple comorbidities, or those with surgical contraindications [33]. The research conducted by Scurtu et al. revealed that a multimodal conservative strategy, integrating cryotherapy, laser ablation, and topical agents, produced outcomes comparable to surgical intervention regarding clearance and recurrence, while being less invasive and more tolerable [24]. Bastos also observed no significant disparities in patient satisfaction, complication rates, or symptom load between the surgical and curettage-electrocoagulation groups, while cosmetic results were marginally superior in the nonsurgical cohort [25]. These findings underscore the necessity for personalized treatment strategies informed by medical risk, lesion attributes, and patient preferences.

Nonsurgical therapies, notwithstanding their potential, possess significant limitations. They frequently need several sessions or prolonged therapy periods, potentially diminishing adherence [22,23]. Furthermore, localized inflammatory responses, particularly with imiquimod or photodynamic therapy, can be unpleasant, visually upsetting, or functionally restrictive, especially in elderly or fragile individuals [23,27]. The lack of histologic proof after nonsurgical therapy raises concerns for residual disease, particularly in tumors with aggressive histologic subtypes [19]. Consequently, although nonsurgical treatments seem appealing for superficial or low-risk basal cell carcinoma, they may not be suitable for all lesion types, especially those exhibiting infiltrative growth patterns or indistinct clinical margins [20,26].

This research's conclusions are consistent with the current literature, notably the 2020 Cochrane review by Thomson et al., which also determined that surgical excision exhibits the lowest recurrence rate and remains the optimal therapy for high-risk BCC [35]. Our evaluation included current trials and included a comprehensive contextual analysis of cosmetic results, patient acceptability, and particular clinical reasons for nonsurgical methods [24,25]. This study highlights the increasing trend in dermatologic oncology towards patient-centered care, focusing on personalized treatment decisions that include clinical effectiveness, patient values, quality of life, and cosmetic results [30].

The mechanistic basis of BCC elucidates its therapeutic response. The majority of basal cell carcinomas (BCCs) are caused by mutations in the hedgehog signaling pathway components, particularly inactivating mutations in patched homolog 1 (PTCH1) and activating mutations in smoothened (SMO), resulting in unregulated cellular growth [6]. Although surgery eliminates altered cells, nonsurgical therapies must address the tumor's inherent resistance mechanisms. Topical and photodynamic therapy rely on limited immune responses or oxidative damage, which may inadequately permeate deeper or more fibrotic tumor areas [27,28]. The extent of lesion invasion, the patient's immunological condition, and the mutation profile may all influence therapy outcomes [29].

The clinical consequences of these discoveries are substantial. For low-risk, superficial basal cell carcinoma, particularly in patients who emphasize cosmetic results or are unsuitable for surgery, topical treatments like photodynamic therapy are effective options [27,29]. Secondly, for nodular or recurring lesions, particularly those exceeding 0.7 mm in depth or exhibiting aggressive histological characteristics, surgical excision is the best method [22,26]. Third, the equilibrium among therapeutic resolution, patient contentment, and esthetic result must be meticulously evaluated, especially in facial or visible regions where scarring may have a prolonged psychological effect [24,25].

Nonetheless, this review possesses some limitations. The variability across the included studies, particularly regarding intervention methods, follow-up duration, outcome measures, and patient demographics, complicates quantitative synthesis of the findings [19,22]. Certain studies were deficient in established instruments for assessing cosmetic results or recurrence, depending instead on subjective evaluations from patients or physicians [25,27]. Furthermore, the majority of research was performed on Western populations, constraining the applicability to those with darker complexion types, disparate healthcare access, or diverse genetic predispositions [19,30].

A notable constraint is the brief follow-up duration in several studies. BCC is a slowly proliferating neoplasm, with recurrences potentially manifesting several years post-initial intervention [16,34]. Most studies documented outcomes between six and 36 months, perhaps failing to reflect long-term treatment effectiveness or recurrence patterns [22,23]. Only a limited number of trials, like Roozeboom et al., have a prolonged follow-up to five years, which should be regarded as the minimal criterion for assessing decisive cancer therapy [28].

Subsequent investigations must rectify these shortcomings by executing extensive, multicenter randomized controlled trials with uniform outcomes, prolonged follow-up periods, and stratification based on basal cell carcinoma subtype and lesion depth [19]. Research should examine the function of molecular markers in forecasting treatment response and assess the application of supplementary imaging modalities such as optical coherence tomography or reflectance confocal microscopy to evaluate the effectiveness of nonsurgical treatments [30]. Cost-effectiveness assessments would assist in directing resource allocation in healthcare systems where patient outcomes and financial sustainability are both critical [25].

This comprehensive analysis affirms that surgical excision remains the most effective and final therapy for BCC, particularly in high-risk or severely infiltrative instances. Nonsurgical techniques, like topical immunotherapy and photodynamic treatment (PDT), are effective in some clinical scenarios, providing similar effectiveness for superficial lesions while yielding superior esthetic results [22,27]. Treatment decisions must be personalized, integrating clinical attributes, patient comorbidities, esthetic preferences, and values to enhance both medical and quality-of-life results [24,30]. An evidence-based, patient-centered strategy is crucial for directing treatment in the changing context of BCC care.

## Conclusions

This systematic review concludes that surgical excision is the gold standard for treating basal cell carcinoma, owing to its superior long-term tumor clearance and lower recurrence rates. However, nonsurgical options, such as topical therapies, photodynamic therapy, and cryosurgery, offer effective and well-tolerated alternatives for specific patient populations, particularly those with superficial lesions, cosmetic concerns, or comorbidities that contraindicate surgery. Treatment selection must be personalized, weighing oncologic efficacy, esthetic results, patient preferences, and general health condition. Future investigations utilizing standardized outcome measures, extended follow-up periods, and stratification by tumor subtype are crucial for enhancing therapy recommendations and facilitating patient-centered decision-making in the management of BCC.
